# Can We Observe Expected Behaviors at Large and Individual Scales for Feed Efficiency-Related Traits Predicted Partly from Milk Mid-Infrared Spectra?

**DOI:** 10.3390/ani10050873

**Published:** 2020-05-18

**Authors:** Lei Zhang, Nicolas Gengler, Frédéric Dehareng, Frédéric Colinet, Eric Froidmont, Hélène Soyeurt

**Affiliations:** 1 TERRA Research Centre, University of Liège-Gembloux Agro-Bio Tech, 5030 Gembloux, Belgium; nicolas.gengler@ulg.ac.be (N.G.); frederic.colinet@uliege.be (F.C.); hsoyeurt@uliege.be (H.S.); 2 Valorisation of Agricultural Products Department, Walloon Agricultural Research Centre, 5030 Gembloux, Belgium; f.dehareng@cra.wallonie.be (F.D.); e.froidmont@cra.wallonie.be (E.F.)

**Keywords:** mid-infrared spectra, milk, feed efficiency, phenotypes

## Abstract

**Simple Summary:**

Feed efficiency and the sustainability of dairy farming are critical concerns for the dairy industry. Studying feed efficiency at a large scale would benefit cow breeding and management on the farm but is unrealistic in the current context due to practical issues. This study used the milk recording database to predict traits related to feed efficiency at large and individual scales in order to observe their behavior throughout the year, and by the lactation stage and parity. This could open new perspectives in the acquisition of traits related to body weight and dry matter intake to develop future selection programs aiming to improve the feed efficiency of dairy cows.

**Abstract:**

Phenotypes related to feed efficiency were predicted from records easily acquired by breeding organizations. A total of 461,036 and 354,148 records were collected from the first and second parity Holstein cows. Equations were applied to the milk mid-infrared spectra to predict the main milk components and coupled with animal characteristics to predict the body weight (pBW). Dry matter intake (pDMI) was predicted from pBW using the National Research Council (NRC) equation. The consumption index (pIC) was estimated from pDMI and fat, and protein corrected milk. All traits were modeled using single trait test-day models. Descriptive statistics were within the expected range. Milk yield, pDMI, and pBW were phenotypically positively related (r ranged from 0.08 to 0.64). As expected, pIC was phenotypically negatively correlated with milk yield (−0.77 and −0.80 for the first and second lactation) and slightly positively correlated with pBW (0.16 and 0.07 for the first and second lactation). Later, parity cows seemed to have a better feed efficiency as they had a lower pIC. Although the prediction accuracy was moderate, the observed behaviors of studied traits by year, stage of lactation, and parity were in agreement with the literature. Moreover, as a genetic component was highlighted (heritability around 0.18), it would be interesting to realize a genetic evaluation of these traits and compare the obtained breeding values with the ones estimated for sires having daughters with reference feed efficiency records.

## 1. Introduction

The theory that life is a chemical process corresponding to metabolism and respiration was first proposed in 1783 [1]. The body has the ability to use nutrients to synthesize tissues [2]. Appropriate nutrient level and management allows the genetic potential of a specific animal to be maximized and the dairy farm sustainability to be improved. Sustainability interrelates different aspects of milk production, from economics (i.e., maximization of milk production per feed quantity), well-being (i.e., knowing the feed efficiency of a cow allows farmers to better feed it and ensures a healthy status), to the environment (such as a lower amount of feed for the same level of milk production leads to less methane eructed by dairy cows) [3]. The improvement of feed efficiency is an opportunity to solve issues facing the dairy industry resulting from production and environmental aspects [4]. Evaluating several traits, like body weight (BW), dry matter intake (DMI), milk production and composition, and consumption index (IC), reflects the feed efficiency of individuals in a certain way [5] and is of interest. This is why most are considered to be important selection traits in animal breeding [6]. 

Feed efficiency can be assessed, for instance, by using the IC defined as the ratio of DMI to fat and protein corrected milk (FPCM) [7]. Although the IC denominator is easily estimated at large and individual scales thanks to routine milk recording, the numerator is harder to record. There have been many attempts to collect this trait, using, for instance, an automated feed monitor, but this recording is limited to a small population of cattle and suffers from inherent difficulty when monitoring pasture-based herds [3]. To solve this problem, the literature proposes an indirect methodology to predict DMI based on more easily recorded traits such as fat corrected milk (FCM), BW, and the week of lactation [8]. FCM and lactation stage are currently documented by all milk recording organizations, but BW is not so frequently recorded. BW can be measured using a weighing scale, predicted using modern technology such as cameras [9], or based on the cow’s morphological traits [10,11,12]. For this last approach, the classification of a cow is unfortunately often realized only once in its life; and due to its cost, the first and second techniques are mainly used in research herds and some high-level commercial herds [13]. Recently, Soyeurt et al. [14] developed an equation to predict an indicator of BW from the milk mid-infrared spectrum (MIR), lactation stage, parity, month of test, and milk yield. The obtained external validation root mean squared error (RMSEPv) ranged from 39 to 64 kg. In conclusion, based on the existing common dairy production traits and using the prediction equations existing for BW and DMI, it is possible to predict several traits related directly or indirectly to feed efficiency at a large scale. This research studies the behavior of the predicted traits with their corresponding reference values published in the literature for several sources of variation such as herd, test day, stage of lactation, and parity. Heritability is also estimated as this genetic parameter reflects the part of the variability transmitted from generation to generation. If the predicted traits available at a large scale depict the expected behavior, those traits could be used in the future to optimize feed efficiency at herd and breeding levels.

## 2. Materials and Methods

This work was carried out in accordance with the EU Directive 2010/63/EU for animal experiments. Milk samples collected for this study were part of the regular milk sampling work undertaken in the Walloon region of Belgium by the Walloon Breeding Association (AWÉ, Ciney, Belgium). Samples were collected from January 2007 until December 2017 from 47,176 first parity Holstein cows in 1204 herds (i.e., 460,765 records, after cleaning) and from 36,208 s parity Holstein cows in 1184 herds (i.e., 353,936 records, after cleaning). Fat (%FAT) and protein (%PROT) contents (g/dL of milk) were determined using FOSS Milkoscan FT6000 spectrometers (Hillerød, Denmark) at the milk laboratory “Comité du Lait” (Battice, Belgium). The generated MIR spectra were also recorded. BW records were predicted (pBW; kg) from those spectra and animal characteristics using the equation developed by Soyeurt et al. [14]. Fat and protein corrected milk (FPCM; kg/d) was calculated using the following formula [15]:

FPCM = Milk yield × (0.337 + 0.116 × %FAT + 0.06 × %PROT)(1)

Then, DMI records at specific test days were predicted (pDMI; kg/day) using pBW, FCM, and the week of lactation using the NRC equation. This equation has a root mean square error of 1.82 kg/day [8] and can be summarized as follows:

pDMI = (0.372 × FCM + 0.0968 × pBW^0.75^) * (1 − exp^(−0.192 × (wol + 3.67))^) (2)

where wol is week of lactation. Fat-corrected milk (FCM; kg/d) was calculated according to the formula [8,16]:

4% FCM = 0.4 × Milk yield + 15 × FAT(3)

Finally, the consumption index (pIC) was predicted as the ratio of pDMI to FPCM. All records fulfilled the following requirements: Records from 5 to 365 days in milk (DIM), pBW between 400 kg and 900 kg [14], milk yield less than 71 kg/d, fat content within the range of 1.5% to 9.0% and protein content ranged from 1.0% to 7.0% [17]. Significance of differences for original predicted traits between parities was assessed using the TTEST procedure in SAS (Version 9.4, SAS Institute, Inc, Cary, NC, USA), as the records were normally distributed and the SD differences for all traits between 2 lactations did not exceed 4 times. Correlation values per lactation were also estimated between studied traits to assess their relationships and for comparisons with the literature. The pedigree related to the first and second parity datasets contained 191,400 animals (180,136 dams and 11,264 sires) and 165,032 animals (154,585 dams and 10,447 sires) born after 1990.

The studied traits were modelled per lactation using 6 univariate test-day models summarized as follows:

y = Xβ + CDIM + QG + Zpe + e(4)

where y was the vector of observations for each trait (i.e., milk yield, %FAT, %PROT, pBW, pDMI, and pIC); β was the vector of herd × test-day (HTD; i.e., 51,334 levels for parity 1 and 50,161 levels for parity 2); DIM was the vector related to the stage of lactation (i.e., 25 classes of 15 DIM); G was the vector of genetic effect (i.e., 191,401 levels for parity 1 and 165,033 levels for parity 2); pe was the vector of permanent environment effect (i.e., 47,175 levels for parity 1 and 36,207 levels for parity 2); e was the vector of residuals. X, C, Q, and Z were the corresponding incidence matrices assigning observations to effects.

The variance components were estimated by the restricted maximum likelihood method (REML) using the REMLF90 program developed by Misztal [18]. Heritability for each studied trait was calculated as the ratio of the genetic variance to the total variance (i.e., the sum of genetic, permanent environmental, and residual variances). Solutions for each effect were estimated using the best linear unbiased prediction (BLUP) method using the BLUPF90 program developed by Misztal [19]. In order to have a cleaned trend, the DIM tendency was assessed using the least squared means (LSMEANS) calculated from the solutions of the DIM effect obtained from the used test-day models. Similarly, the test month effect was studied by averaging per month LMEANS estimated from the HTD solutions.

## 3. Results

### 3.1. Parity Effect

The pBW of the second lactation was significantly higher than the first lactation, and the difference was 42 kg (Table 1). A significant increase occurred for pDMI between the first and second lactations (19.45 to 21.16 kg/day, Table 1). The global mean of pDMI for the first and second lactation was 20.30 kg/day. A positive difference of 3.09 kg/day was observed for milk yield between the first and second lactation. The contents of fat and protein in the first lactation were 3.98 and 3.38 g/dL of milk, which were lower than the ones observed in the second lactation (4.07 and 3.46 g/dL of milk). The pIC decreased significantly from the first lactation (0.87) to the second lactation (0.84).

### 3.2. Effect of Lactation Stage

**Figure 1:**
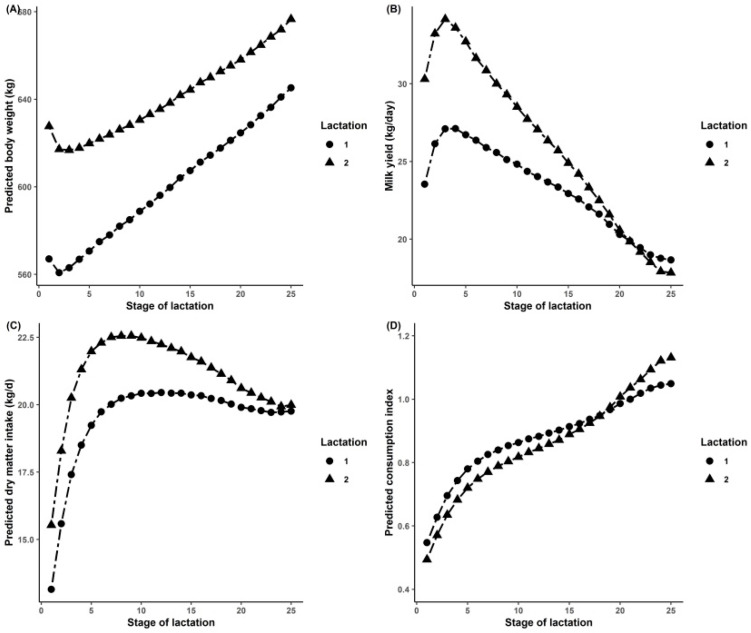
Evolution of predicted body weight (**A**), milk yield (**B**), predicted dry matter intake (**C**), and predicted consumption index (**D**) throughout lactation.

The evolution of initial predicted traits and milk yield are shown in Figure 1. The interest of this paragraph is to ensure that the predicted traits used in this study followed the expected trends. The pBW dropped to a lower point after calving and then increased slightly. The pDMI got a top peak around 2 months after calving and then decreased slowly. The peak of pDMI appeared later than the appearance of the milk peak. In brief, milk yield increased sharply compared to pDMI at the beginning of lactation, involving a negative energy balance and a loss of weight (i.e., pBW in this study). A smooth slope appears in the pIC curve, which means that the feed efficiency turned worse gently at that time.

### 3.3. Correlations between Studied Traits

As shown in Table 2, the first and second parity Holstein cows showed different correlation values estimated between all studied traits. The pBW was slightly positively correlated with milk yield for both lactations (r = 0.09 or 0.08). The pDMI was also positively correlated with milk yield and pBW. There were positive relationships between pIC and fat or protein contents in milk (Table 2). A similar correlation value was observed between milk yield and pIC (Table 2). In this study, we found a negative correlation between pIC and pDMI (r = −0.17 and −0.38), but this relationship was weaker compared to the one observed with milk yield (r = −0.77 and −0.80). Slightly positive relationships were found between pBW and pIC (0.16 and 0.08 for parity 1 and 2).

### 3.4. Test Month Influence

**Figure 2:**
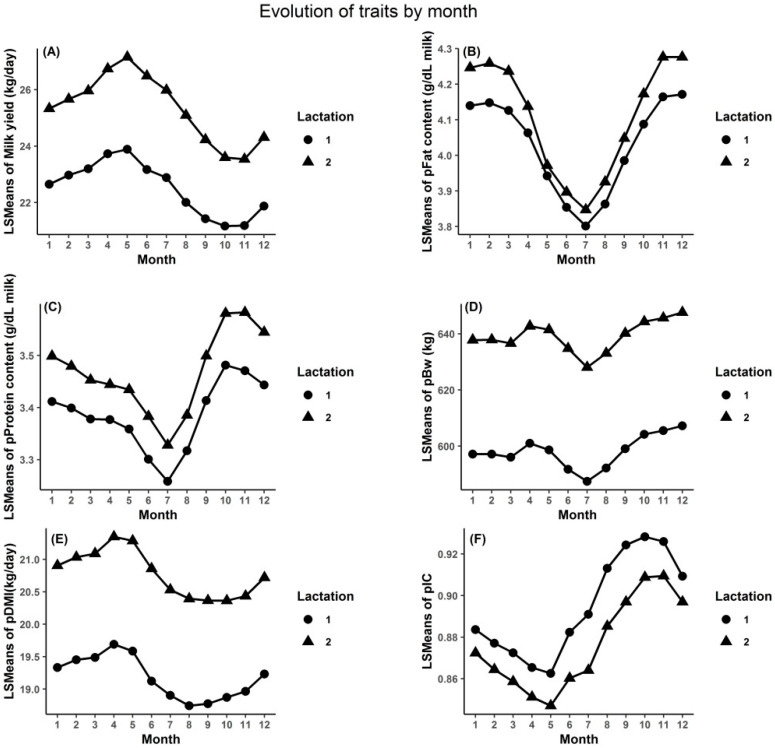
The evolution of the averaged herd least square means (LSMEANS) for all studied traits across test months (LSMEANS of milk yield (**A**); LSMEANS of predicted fat content (**B**); LSMEANS of predicted protein content (**C**); LSMEANS of predicted body weight (**D**); LSMEANS of predicted dry matter intake (**E**); LSMEANS of predicted consumption index (**F**)).

The averaged herd LSMEANS varied throughout the year for all studied traits and for both lactations studied (Figure 2). As observed previously from raw data, the second lactation showed a higher milk yield, fat and protein content, pBW, and pDMI compared to the first lactation. There were 3 kinds of seasonal trends for the studied traits: (1) Milk yield and pDMI; (2) %PROT, %FAT, and pBW; (3) pIC. There was a slight increase in milk yield and pDMI until May, then a smooth decrease, and another increase in August and November for pDMI and milk, respectively. The %PROT, %FAT, and pBW showed similar annual behavior. Specifically, pBW was mostly stable over time, showing a slight drop during the summertime and then an increase. The %PROT was lower in July (i.e., 3.26 g/dL ± 0.20 g/dL of milk for first lactation and 3.33% ± 0.21% for the second lactation, respectively), and reached a peak in October (Figure 2). This tendency was analogous to the trend in %FAT, except that this trait had a peak in November and December, which was approximately 1 month later compared to %PROT. The evolution of pIC for both studied lactations dropped smoothly until May and then peaked in November, which was quite contrary to the annual trend observed for milk yield. The best feed efficiency did not occur at the point of the highest milk yield or the lowest DMI.

### 3.5. Heritability

The estimated heritability of pDMI was 0.14 and 0.11 for the first and second lactation. The heritability estimates for pBW were also moderate and equal to 0.18 and 0.17 for the first and second lactation. Moderate heritability values were also estimated for pIC (0.14 and 0.09 for first and second lactation).

## 4. Discussion

### 4.1. Parity Effect

The growth of a cow directly impacts its morphology and, therefore, the body weight. Thus, a positive BW difference is expected between primiparous and multiparous dairy cows. Although BW was predicted using MIR spectrometry and animal characteristics, the trend observed for this predicted phenotype (Table 1) is in agreement with the behavior observed for the reference measured trait. Indeed, the same trend was reported by Ferris et al. [20], who found reference BW for Holstein-Friesian cows for the first lactation to subsequent lactations ranged from 588 kg to 702 kg.

The BW is not the only morphological trait influenced by growth; the whole body of the cow is impacted. These changes in morphological traits induce an increase in the capacity for feed ingestion by the cow. Thus, more concretely, a significant positive difference between the first and second parity was, therefore, expected for DMI and also for the predicted trait related to DMI, as is the case in this study. The global mean of pDMI was similar to the DMI value reported by De Boever et al. [21]. They observed an average DMI of 20.87 kg/day for Holstein-Friesian cows at an average lactation of 2.3.

Moreover, growth and related morphological changes also indirectly impact the quantity and the composition of milk. Less mature cows tend to use their limited nutrition intake to complete their own physical development rather than producing more milk [22,23]. This happens alongside growth until the fourth or fifth lactation [24,25,26]. Moreover, the volume of the mammary gland increases with growth and this is positively correlated to the milk yield [27]. Thus, all of these things can explain the positive difference of 3.09 kg/day observed for milk yield in this study (similar to the finding of 3.0 kg/d made by Davis et al. [27]) as well as the lower amount of fat and protein produced in it. Younger cows need more nutrients to fulfill body growth and, therefore, produce milk with a lower content of fat and protein [28]. Such differences in milk fat and protein contents between the first and second parities were also observed by other authors. For instance, Craninx et al. [29] observed 3.83% and 4.30% fat from primiparous and multiparous cows, respectively. Morales-Almaráz et al. [30] measured 3.66% and 3.93% of fat in milk produced by primiparous and multiparous non-grazing cows, respectively. Thus, there is a link between morphological traits and dairy production phenotypes. This is why the calculation of the consumption index expressed as the ratio of an indirectly related morphological trait, like DMI, to the milk production trait (FPCM) is of interest to assess feed efficiency. Theoretically, the most productive cows will be the healthy one, which can be able to maximize the production of milk for a given amount of feed. As a basis, the cows’ genetic background of feed utilization must be taken into consideration. In the field, farmers also need to balance the feed efficiency and the veterinarian cost of a cow to enhance the sustainability of a farm.

The difference of pIC between lactations means that the cows in the second lactation seemed to be more efficient than the cows in the first lactation. This finding is biologically relevant as primiparous cows tend to use nutrients to grow rather than to produce milk [23]. However, Oldenbroek et al. [31] studied different breeds of cow and found there was no significant effect of parity on the ratio of milk energy production to the net energy intake, but the cows in second parity were more efficient than the cows in first parity. This conclusion was similar to the trend observed in the present study.

### 4.2. Effect of Lactation Stage

We know that growth, as well as the gestation of the cow, can impact the body weight and also the quantity and the composition of milk. This can be observed by checking the evolution of traits throughout lactation (Figure 1). It is interesting to note that the curves for pBW and pDMI were similar to those presented in the studies [32,33] conducted on Holstein-Friesian cows for BW and DMI up to 9 weeks after calving. Another study [34] also concluded a similar curve of DMI within lactation from dairy cows from nine different countries. However, their DMI curve decreased more gently compared to the pDMI trend observed in the present study. Logically, the observed trends can be explained again by the concept of nutrient allocation. A study reported that new calving dairy cows cannot maintain a positive dietary energy balance and must mobilize body reserves to compensate for the negative energy balance [35]. By contrast, pDMI increased from calving until the mid-lactation, therefore, increasing the quantity of available energy and leading to an increase of BW. Thus, pBW increased after the slight drop in early lactation. From reference measurements, Ntallaris et al. [36] reported a significant increase of DMI during the first 120 days postpartum in Holstein cows. The research reported that energy equilibrium for first and older lactating cows was reached at week 13 and week 18 of lactation, respectively [37]. This could explain why pDMI had a peak at early lactation. The peak delay of pDMI compared to milk peak is consistent with the results reported by Hristov et al. [38], supporting the theory that the feed intake is driven by milk production [8].

For the smooth slope of the pIC curve, it can be explained by the fact that the feed efficiency-related traits like FPCM and DMI were not constant across the whole lactation period [39].

### 4.3. Correlations between Studied Traits

A positive relationship between pDMI with milk yield was observed in the current study (Table 2). A similar positive relationship between these traits (r = 0.57 for DMI and milk yield) was observed from reference measurements [40]. Similarly, DMI increased with milk yield and BW in Holstein-Friesian cows [41]. Although there were not many reports on correlations between BW and daily milk yield, the absence of a relationship between BW and milk yield was observed from Holstein (N = 1584) and Jersey (N = 679) cows and the reason may be related to the health status of the cows [42]. A positive correlation (r = 0.40) between metabolic BW and DMI was observed [40]. Asher et al. [43] reported a correlation coefficient equal to 0.26 for Israeli Holstein cows between BW and DMI, but this value was not significant. Those authors concluded that the positive correlation was due to the biological increase of feed digestibility. However, the lack of significance (*p* = 0.14) observed by those authors may be related to the size of the dataset (N = 35); a larger cohort could produce a different result.

Positive relationships between pIC and fat or protein contents were observed in this study. Similarly, another study [40] reported that high feed efficiency cows produced lower fat and protein contents in milk compared to low feed efficiency cows. This is related to the fact that a cow with high feed efficiency has higher milk production, which explains why a negative correlation between milk yield and pIC is expected. The observed similar correlation value between milk yield and pIC is in agreement with the conclusion of Kover [44], who stated that high producing cows had a better feed efficiency. Meir et al. [40] reported that the ratio of ECM to DMI (i.e., another expression of feed efficiency) in Holstein cows was positively correlated with milk yield but negatively correlated with DMI. Similarly, a positive correlation (r = 0.18) between feed efficiency (defined as FCM divided by energy intake) and energy intake was reported suggesting that the variation in feed efficiency depends more on the milk production than the feed consumption [45].

The negative correlation between pIC and pDMI is a consequence related to the positive relationships existing between pDMI and milk yield. Moreover, as the animal satisfies first its maintenance (and growth) requirements essentially. Afterward, it uses nutrients better for milk production, and this is known that above a certain limit, the marginal use of nutrients for milk production decreases. Therefore, there may be a curvilinear relation between pIC and pDMI. A part of this positive correlation is also related to the fact that pDMI includes in its formula the information about milk yield.

The slightly positive relationships between pBW and pIC mean that heavier cows would tend to present lower feed efficiency. This statement was also expressed in the past [46]. A negative correlation (r = −0.20) was reported between BW and feed conversion efficiency (DMIE, defined as 305-d FCM divided by 305-d DMI) [47]. This study confirmed the positive value observed in the current research as we have used an inverse indicator of feed efficiency. The low value of correlation was expected based on the findings [42], which observed a quadratic relationship between feed efficiency with the BW in the first lactation of Holstein cows, and the correlation coefficient may also change.

### 4.4. Test Month Influence

The trends of %PROT (Figure 2) is in agreement with the findings from previous researches [48]. Those authors also found that the %FAT and %PROT dropped during the summertime and then slightly increased. The gentle increase in pBW observed in the current study, during the period around winter when concentrate supplement is given to cows, was consistent with results reported previously [49].

Holstein cows showed lower feed efficiency in warm seasons than cold seasons [50]. In the present study, the findings suggest that the dairy cows had higher feed efficiency around the springtime when the temperature was comfortable and concentrate supplementation was available.

Different reasons can explain the three trends observed. The first reason and the main one is the change in diet given to the cows throughout the year. Feed rations richer in concentrates are currently given to cows during the non-grazing time (i.e., the winter season), leading to increased DMI, milk yield, and %PROT [49,51]. Some authors have found that milk fat has an increasing trend when a higher amount of concentrate is given to the cow (70% vs. 50% DM) [52,53], but this effect depends heavily on the composition of the concentrate, which is related to changes in the ratio of acetate to propionate in the rumen [54]. In the current study, higher milk fat contents were observed during the winter period (i.e., when concentrate supplements were given to the cows), and the milk yield was relatively low. The second reason, coupled to the first one, is the physical activity of the cows. During the grazing period, the cows will have a higher physical activity, which impacts the energy partitioning and may lead to a decrease of milk production and BW. The third reason for annual changes observed during the summer is that high temperatures relate to the discomfort of the cow living in this environment [55]. During this time, the cow will eat less (i.e., lower DMI) due to heat stress, negatively impacting milk production [56] and potentially also BW [57].

### 4.5. Heritability

The estimated heritability of pDMI (Table 3) was similar to the value of 0.17 reported before for DMI [47]. This is also within the range of heritability for DMI proposed by Hardie [58] for dairy cows (from 0.02 to 0.52). The heritability of pBW is similar to the value of 0.19 [59] and within the range proposed in research for the BW of Holstein cows (from 0.20 to 0.80) [45]. The heritability of pIC was moderate. Generally, the heritability of gross feed efficiency ranges from 0.12 to 0.63 for dairy cows [44]. These values were in agreement with those estimated by other authors for other traits related to feed efficiency. A value of 0.18 for feed efficiency defined as 305-d FCM divided by net-energy intake for 970 dairy cows was reported [47]. Thus, heritability estimates of pBW, pDMI, and pIC were moderate and slightly lower for the second lactation than the first lactation (Table 3). A similar trend was found [60] in which the 305-d DMI of cows in the third parity was 0.08, lower than the 0.12 for cows in first parity, and concluded this may be due to the ongoing breeding selection of cows, leading to a lower genetic variance for mature cows.

## 5. Conclusions

Due to practical issues, measuring traits related directly or indirectly to feed efficiency, such as BW, IC, and DMI at large and individual scales, is difficult, if not impossible. However, predicting those traits using animal production characteristics and MIR spectrometry is feasible. However, as it was not possible to conduct a large scale validation of these predictions, this study aimed to predict these traits using the Walloon milk recording database in order to observe the behavior of the traits in real conditions and compared them to findings reported in the literature. The results revealed that behavior throughout the year, the stage of lactation, and the parity for these predicted traits fully agreed with the expected values, suggesting that relevant information was provided by the traits, even if their prediction accuracy was moderate. Additionally, these traits presented moderate genetic variability. All of these aspects suggest the potential interest of using predicted traits to develop management and breeding tools. To confirm this interest, a large scale study may be conducted to assess the accuracy of predictions. For that, it will be relevant to combine the efforts made internationally in many research farms where the routine acquisition of DMI and BW is possible. This could allow comparisons of the prediction with real reference values, collected in many different conditions, in order to establish variability that is representative of the dairy cow population. Moreover, as a genetic component exists, it could be interesting to realize a genetic evaluation of the feed efficiency-related traits and compare the breeding values estimated for sires having daughters with predicted and reference records. If the ranking of bulls is similar, this will open new perspectives on the use of these traits to develop future selection programs aiming to improve the feed efficiency of dairy cows.

## Figures and Tables

**Table 1:** Descriptive statistics of the studied datasets.

Traits	Mean ± SD ^1^		Minimum	Maximum
	Lactation 1	Lactation 2		
Milk yield (g/day)	23.54 ± 6.00 ^B^	26.63 ± 8.19 ^A^	3.10	70.60
Fat content (g/dL of milk)	3.98 ± 0.69 ^B^	4.07 ± 0.73 ^A^	1.50	9.00
Protein content (g/dL of milk)	3.38 ± 0.36 ^B^	3.46 ± 0.39 ^A^	1.19	7.00
Predicted body weight (kg)	597 ± 35 ^B^	639 ± 31 ^A^	421	820
Predicted dry matter intake (kg/day)	19.45 ± 2.71 ^B^	21.16± 2.92 ^A^	7.50	39.21
Predicted consumption index	0.87 ± 0.18 ^A^	0.84 ± 0.22 ^B^	0.33	4.98

^1,A,B^ Means within a row with different superscripts differ significantly from each other (*p* < 0.001).

**Table 2:** Correlation coefficients observed between studied traits in first (below the diagonal) and second lactation (above the diagonal).

Trait *	Milk	%FAT	%PROT	pBW	pDMI	pIC
Milk yield (kg/day)		−0.37	−0.50	0.08	0.64	−0.80
Fat content (g/dL of milk; %FAT)	−0.35		0.54	0.13	−0.06	0.11
Protein content (g/dL of milk; %PROT)	−0.35	0.49		0.51	−0.12	0.37
Predicted body weight (kg; pBW)	0.09	0.16	0.61		0.47	0.08
Predicted dry matter intake (kg/day; pDMI)	0.59	0.01	0.15	0.62		−0.38
Predicted consumption index (pIC)	−0.77	0.08	0.31	0.16	−0.17	

* The correlations for all traits were significant (*p* < 0.001).

**Table 3:** Heritability (h^2^) of studied traits for the first two lactations.

Traits	Heritability	
	Lactation 1	Lactation 2
Milk yield	0.20	0.16
%FAT	0.37	0.41
%PROT	0.41	0.40
pBW	0.18	0.17
pDMI	0.14	0.11
pIC	0.14	0.09

## Abbreviations

The following abbreviations are used in this manuscript (in alphabetical order):

%FAT	Fat contents
%PROT	Protein contents
BLUP	Best linear unbiased prediction
DIM	Days in milk
ECM	Energy corrected milk yield
FCM	4%Fat corrected milk yield
FPCM	Fat and protein corrected milk yield
HTD	Herd test day model
LSMEANS	Least square means
MIR	Mid-infrared spectrum
NRC	National Research Council
pBW	Predicted body weight
pDMI	Predicted dry matter intake
pIC	Predicted consumption index
RMSEPv	Root mean square error of validation
